# SREBP activation contributes to fatty acid accumulations in necroptosis†

**DOI:** 10.1039/d2cb00172a

**Published:** 2023-02-13

**Authors:** Daniel Lu, Laura R. Parisi, Omer Gokcumen, G. Ekin Atilla-Gokcumen

**Affiliations:** a Department of Chemistry, University at Buffalo, The State University of New York (SUNY) Buffalo NY 14260 USA ekinatil@buffalo.edu; b Department of Biological Sciences, University at Buffalo, The State University of New York (SUNY) Buffalo NY 14260 USA

## Abstract

Necroptosis is a type of programmed cell death. It is characterized by membrane permeabilization and is associated with the release of intracellular components due to compromised membrane integrity which induces a strong inflammatory response. We recently showed that the accumulation of very long chain fatty acids (VLCFAs) contributes to membrane permeabilization during necroptosis. However, the mechanisms that result in the accumulation of these cytotoxic lipids remain unknown. Using comparative transcriptomics and digital PCR validations, we found that several target genes of sterol regulatory element-binding proteins (SREBPs) were upregulated during necroptosis, suggesting that they might be responsible for the accumulation of VLCFA in this process. We demonstrated that activation of SREBPs during necroptosis exacerbates the permeability of the plasma membrane and cell death. Consistent with these observations, targeting sterol regulatory element-binding protein cleavage-activating protein (SCAP), a protein involved in SREBP activation, reversed the accumulation of VLCFAs, and restored cell death and membrane permeabilization during necroptosis. Collectively, our results highlight a role for SREBP in regulating lipid changes during necroptosis and suggest SREBP-mediated lipid remodeling as a potential target for therapeutics to reduce membrane permeabilization during necroptosis.

## Abstract 2

.Necroptosis is accompanied by accumulation of lipids. This study shows that SREBPs are activated and cause increase in the production of very long-chain fatty acids during necroptosis.

## Graphical abstract

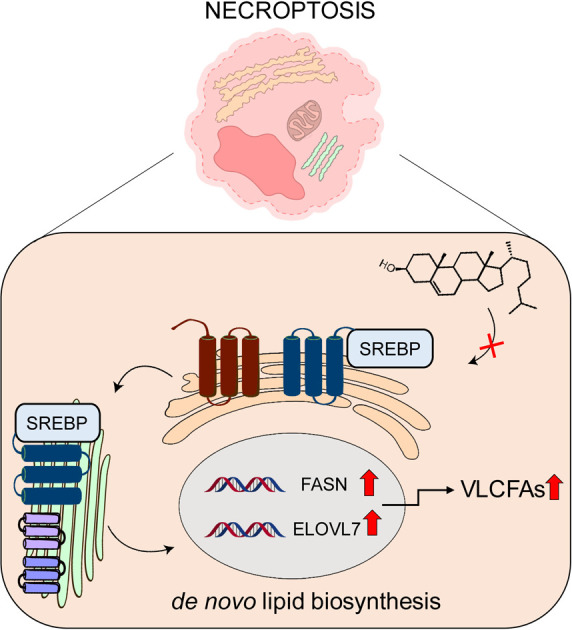

## Introduction

Lipids have a plethora of cellular functions. They provide structural integrity for biological membranes^1^ and actively participate in cellular processes, including cell death and survival.^2^ At the intersection of their structural and signaling roles, lipids participate in membrane-related transformations that occur during different cellular processes.^3–5^ For instance, cells can modulate lipid production to provide key metabolites needed for sustained proliferation rates^6,7^ and suppress the biosynthesis of cell survival-related lipids.^8^ Despite these key roles in survival, dysregulated accumulation of lipids can trigger lipotoxicity which contributes to many cellular fates.^9,10^ In many pathologies, the increased lipid uptake or imbalance of cellular lipolytic and lipogenic activities lead to the accumulation of lipids that cause organelle and membrane damage, perturb fatty acid breakdown, and eventually lead to cell death.^11,12^ One of the major mechanisms that regulate *de novo* lipid biosynthesis is the transcriptional activation of sterol regulatory elementary binding proteins (SREBPs). SREBP1 and SREBP2, encoded by two different genes, regulate the production of fatty acids and sterols, respectively.^13^ SREBP transcriptional activity is regulated by a metabolite-driven feedback loop. At the endoplasmic reticulum (ER) membrane, SREBP forms a complex with SREBP cleavage-activating protein (SCAP) under reduced cholesterol conditions. The SCAP-SREBP complex exits the ER *via* endocytosis, translocates to the Golgi, and is proteolytically cleaved, releasing the mature transcriptionally active form – matur SREBP – which then translocates into the nucleus where it binds to genes involved in lipid biosynthesis and transport. Hence, the interaction between the SCAP-SREBP complex is critical for the transcriptional activation of SREBP target genes. When present at high levels in the ER, cholesterol binds to SCAP and prevents its interaction with SREBP, inhibiting SREBP from leaving ER, thus downregulating lipid production. In parallel to the canonical feedback inhibition of SREBP, other possible cholesterol-independent regulations of SREBP in cells are discovered. Recent studies reported that SREBPs can undergo activation *via* extracellular matrix mechanical cues and acto-myosin contractility.^14,15^ Another study also showed SRBEP activity may be sensitive toward acidic pH in the extracellular environment.^16^

Necroptosis, also known as programmed necrosis, is a form of non-apoptotic cell death.^17,18^ It is associated with many diseases, including ischemia/reperfusion injuries,^19^ myocardial infarction,^20^ and cancer.^21^ Importantly, necroptotic cell death contributes to the inflammatory responses associated with these diseases due to the permeabilization of the plasma membrane upon activation of the death pathway.^22^ The permeabilization of the plasma membrane, in particular, results in the release of cellular content, including cytokines, to the extracellular matrix, which induces an inflammatory response at the organismal level.^23,24^ Blocking necroptosis has provided encouraging results in reducing inflammation in psoriasis^25^ and delaying the loss of motor function in a model of Huntington's disease.^26^ In cancer therapeutics, necroptosis is often viewed as a double-edged sword. While functioning as a fail-safe cell death mechanism in apoptosis-resistant cancer cell lines has been shown as a promising strategy, the increase of downstream inflammation and other immune response during necroptosis plays a paradoxical role in tumor progression.^27^ Upon membrane permeabilization, intracellular components including pro-inflammatory cytokines, chemokines, and other growth factors are released into the microenvironment, promoting tumorigenesis.^28^ It has been reported that necroptosis can promote the production of pro-inflammatory cytokines^29^ and cancer metastasis in the tumor microenvironment.^30^

Targeting necroptosis, specifically reducing the release of pro-inflammatory stimulants *via* membrane permeabilization, has become a potential therapeutic strategy in combating tumor progression. For instance, a study has demonstrated that the suppression of necroptosis *via* small molecule inhibitor necrostatin-1s successfully reduces tumor growth in the colitis-associated tumorigenesis mouse model.^31^ Another study has shown inhibition of necroptosis either by small molecule inhibitors or deletion of key proteins in necroptosis activation, reducing endothelial necroptosis and metastasis.^32^ As such cancer progression and metastasis may be linked to the downstream release of inflammatory substances as a result of membrane permeabilization during necroptosis. Thus, specific targets that contribute to membrane permeabilization and toxicity during necroptosis are an exciting frontier for new therapeutics.

The molecular interactions that cause the disintegration of the plasma membrane during necroptosis are well-studied. The membrane modeling that eventually results in membrane rupture is dependent on the activity of Receptor Interacting Protein Kinases 1 and 3 (RIPK1/RIPK3).^17,33^ Upon activation of death pathways, a complex consisting of RIPK1, RIPK3, and Mixed Lineage Kinase Domain-like protein (MLKL) forms in necroptosis.^34^ This complex phosphorylates MLKL and facilitates the oligomerization of MLKL and its subsequent translocation to the plasma membrane, which compromises the plasma membrane integrity.^35^ Translocation of phosphoMLKL (pMLKL) oligomers to the plasma membrane are primarily driven by electrostatic interactions between the oligomer interface and negatively charged phosphatidylinositol phosphate (PIP)-rich membrane domains.^36^ In parallel, recent studies from our group have demonstrated that *S*-fatty acylation of pMLKL by very-long-chain-fatty acids (VLCFAs, fatty acids >20 carbons^37^) help membrane binding of pMLKL and contribute to membrane permeabilization during necroptosis.^38^ Overall, these two chemical interactions, electrostatic and acylation-mediated membrane binding, cause pMLKL to locate in the plasma membrane and result in the consequent pore formation, which is the basis for the inflammatory nature of necroptotic cell death.^35,36^ These findings clearly suggest critical involvement of lipids and membranes in necroptosis. However, regulatory mechanisms that govern lipid changes in necroptosis are unknown.

In our recent studies toward a better understanding of the contribution of lipids to membrane permeabilization during necroptosis, we found that lipids accumulated during this process.^39^ Specifically, we found that VLCFAs showed profound accumulations at the transcriptional level during this process *via* the activation of fatty acid synthase (*FASN*) and the elongation of very long chain fatty acids proteins 1 and 7 (*ELOVL1* and *ELOVL7*). We further showed that pMLKL and MLKL, key signaling proteins of necroptosis, undergo fatty acylation by VLCFAs, which improves their membrane recruitment and binding and contributes to plasma membrane permeabilization during necroptosis.^38,40^

In this work, we investigated the mechanisms of activation of lipid production in necroptosis. Our transcriptomics analyses suggested that SREBPs and their downstream targets induce the accumulation of VLCFA during necroptosis. We verified these findings by showing that activation of SREBPs exacerbates cell death. In contrast, inactivating them reduced cell death and significantly restored membrane permeabilization. Overall, our results provide a better understanding of the mechanism of necroptosis and pave the way to prioritize lipid-related pathways for therapeutic strategies.

## Results

### Transcriptomic analysis highlights the upregulation of lipid biosynthesis-related genes

To investigate the pathways responsible for the transcriptional activation of *de novo* VLCFA biosynthesis during necroptosis, we compared the transcriptomes of necroptotic and control HT-29 cells. Necroptosis was induced as we described previously.^38–40^ Briefly, HT-29 cells were first pretreated with small molecule inhibitors BV6^41^ (antagonist of the inhibitor of apoptosis proteins) and zVAD-fmk^42^ (a pan-caspase inhibitor) that sensitizes cells towards TNF-induced necroptotic cell death pathway. Necroptosis was then initiated *via* the addition of TNF-α. Control and necroptotic cells were collected and RNA was extracted (*n* = 3 for each condition). The mRNA content was analyzed using Illumina sequencing (see Methods section for details).

**Fig. 1:**
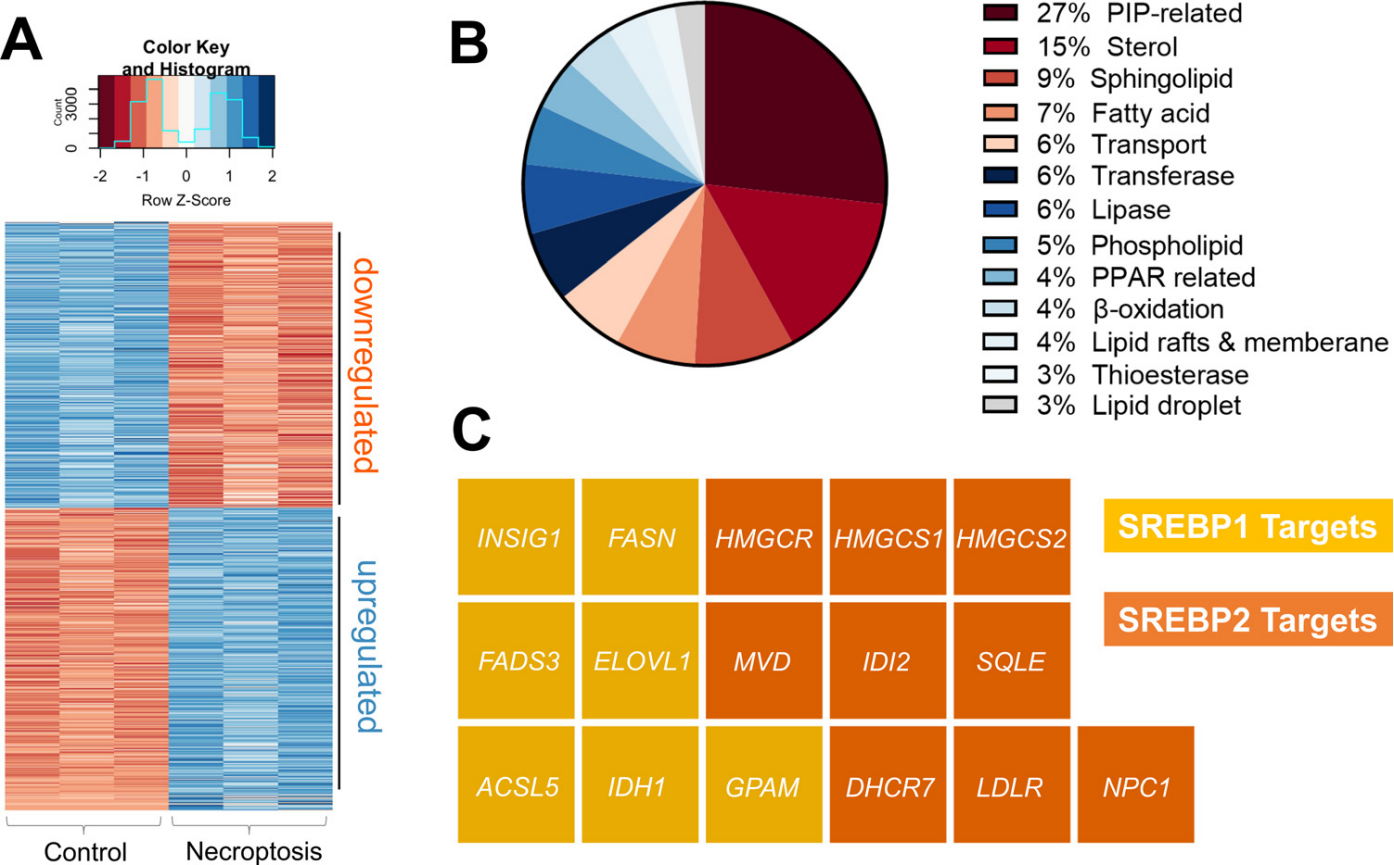
Transcriptomics analysis during necroptosis. (A) Heatmap representation of transcripts that are differentially regulated during necroptosis (*p*_adjusted_ < 0.05) (B) Among the differentially regulated transcripts, 113 were lipid production, processing, and transport-related. The pie chart shows the distribution of these 113 transcripts based on their function. (C) Waffle plot of differentially regulated SREBP targets during necroptosis. Genes of interest were normalized based on the gene expression level in control cells and the fold change was calculated based on the ratio between the average of necroptotic cells with respect to control cells. Genes abbreviation: insulin-induced gene 1, *INSIG1*; fatty acid synthase, *FASN*; fatty acid desaturase 3, *FADS3*; ELOVL fatty acid elongase 1, *ELOVL1*; acyl-CoA synthetase long-chain family member 5, *ACSL5*; isocitrate dehydrogenase 1, *IDH1*; glycerol-3-phosphate acyltransferase, *GPAM*; isopentenyl-diphosphate delta isomerase 2, *IDI2*; low density lipoprotein receptor, *LDLR*; 3-hydroxy-3-methylglutaryl-CoA synthase 1, *HMGCS1*; Niemann-Pick disease, type C1, *NPC1*; squalene epoxidase, *SQLE*; mevalonate (diphospho) decarboxylase, *MVD*; 7-dehydrocholesterol reductase, *DHCR7*; 3-hydroxy-3-methylglutaryl-CoA synthase 2, *HMGCS2*; 3-hydroxy-3-methylglutaryl-CoA reductase, HMGCR.

By comparative analysis, we identified genes that show differential expression between control and necroptotic cells (Table S1, ESI†*p*_adjusted_ < 0.05). Fig. 1(A) represents 3174 genes (1631 upregulated and 1543 downregulated in necroptotic conditions). After manual examination of these genes, we found that 113 of these were lipid-related (Fig. 1(B) and Table S2, ESI†). We validated these findings by measuring the changes in gene expression in an independent set of samples using digital droplet PCR (Fig. S1, see Table S3, ESI† for primer sequences). Among the lipid-related transcripts that were regulated during necroptosis, we noticed several major targets of SREBP1 and 2. These included key enzymes that regulate *de novo* fatty acid (*e.g.*, fatty acid synthase, *FASN*; elongation of very long chain fatty acids protein 1, *ELOVL1*)^43,44^ and cholesterol biosynthesis (*e.g.*, 3-hydroxy-3 methylglutaryl-CoA reductase, *HMGCR*; squalene epoxidase, *SQLE*)^43^ (Fig. 1(C)). These observations prompted us to investigate the potential role of SREBPs in regulating lipid composition during necroptosis.

### SREBPs are activated during necroptosis

**Fig. 2:**
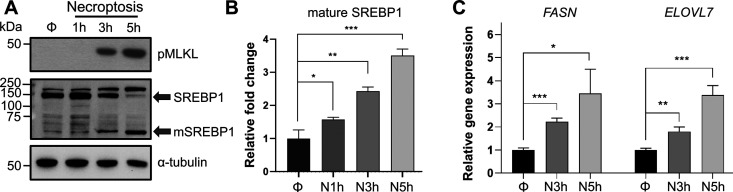
SREBP1 is activated during necroptosis. (A) and (B) Western blot analysis for the time-dependent increase in SREBP1 activation during necroptosis in HT-29 cells. Necroptosis was induced for 1 h, 3 h, and 5 h. The whole lysate samples were prepared and analyzed. Transcriptional active fragment, mature SREBP1 (mSREBP1) increases time-dependently compared to control during necroptosis. (A) A representative western blot is shown. (B) Quantification of mSREBP1 band intensities shows a significant increase during necroptosis compared to control cells. *Φ* represents DMSO control, N1 h represents 1 h necroptosis, N3 h represents 3 h necroptosis and N5 h represents 5 h necroptosis. Data represent mean ± 1 standard deviation (SD); *n* = 3. * represents *p* < 0.05, ** represents *p* < 0.01, *** represents *p* < 0.001. (C) SREBP1 target genes showed upregulation during necroptosis in HT-29 cells. Fold changes in the expression of *FASN* and *ELOVL7*, are calculated as the ratio of relative expression of each gene compared with *HPRT1* in necroptotic and control cells. Data represent mean ± 1 SD; *n* = 3. * represents *p* < 0.05, ** represents *p* < 0.01, *** represents *p* < 0.001.

Transcriptional regulation of *de novo* lipid biosynthesis by SREBP1 and 2 is one of the major mechanisms that control lipid production in cells.^43^ SREBP1 primarily activates the transcription of genes responsible for the biosynthesis of fatty acids.^45^ SREBP2, on the other hand, activates genes responsible for cholesterol biosynthesis and uptake.^13^ It is important to note that previous studies *in vitro* and *in vivo* have pointed towards a complex crosstalk between SREBP1 and 2 activity where knockdown or knockout of either of these isoforms are compensated at the lipid synthesis level *via* activation of the other isoform or other lipid regulatory mechanisms.^46–48^ Because we were interested in the accumulation of VLCFAs during necroptosis, we initially focused on the activation of SREBP1. To investigate the activation of SREBP1 and the effect of this activation on lipid targets during necroptosis, we first studied its proteolytic maturation using Western blotting. HT-29 cells were pretreated with BV6 and zVAD-fmk for 30 minutes and necroptosis was induced *via* the addition of TNF-α for 1 h, 3 h, or 5 h, as we reported previously.^38–40^ Using Western blotting we observed a time-dependent increase in mature SREBP1 levels (up to 3.5 fold, *p* < 0.001) during necroptosis (Fig. 2(A) and (B)). Next, to investigate the effect of SREBP1 maturation on target genes, we used digital droplet PCR and quantified the changes in *FASN* and *ELOVL7* expression, representative SREBP1 targets that control fatty acid synthesis, as necroptosis progressed. Similar to our observation of the time-dependent activation of SREBP1 during necroptosis, the levels of FASN and ELOVL7 increased up to 4-fold during necroptosis (Fig. 2(C)). We note that the activation of SREBP1 during necroptosis is not specific to HT-29 cells. We induced necroptosis in U937 cell line (human histiocytic lymphoma cell line) as we described previously^39^ (Fig. S2A, ESI†) and showed a time-dependent increase in mature SREBP1 levels (Fig. S2B, ESI†), similar to our observations in HT-29 cells. These results show that SREBP1 is activated independent of cell type during necroptosis.

We also observed similar activation of SREBP2 during necroptosis in HT-29 (Fig. S3A, ESI†) and U937 cells (Fig. S2B, ESI†) indicated by the increases in the expression of its target genes (Fig. S3B, ESI†). To investigate if SREBP2 activation could be involved in lipid regulation during necroptosis, we measured cellular cholesterol levels in control and necroptotic cells using LC–MS as we described earlier.^39^ There was no appreciable change in cholesterol levels during necroptosis (Fig. S3C, ESI†), suggesting that SREBP2 activation does not affect cholesterol pools in necroptosis. Further, we used several small molecule inhibitors of the cholesterol biosynthetic pathway (Fig. S3D, ESI†) and investigated the effect of these inhibitors on cell viability during necroptosis. Based on the dose-response curves we generated (data not shown), we chose the highest concentration for each inhibitor that had no significant effect on cell viability on their own and measured cell viability during necroptosis in the presence of these inhibitors. None of the inhibitors we tested significantly affected cell death during necroptosis (Fig. S3D, ESI†), suggesting that activation of SREBP2 does not affect cell death during necroptosis.

TNF-α may stimulate the activation of SREBP1 in human hepatocytes independent of sterol levels, suggesting a direct role of TNF signaling in SREBP1 activation.^49^ To test if the SREBP1 activation we see in necroptosis is also due to TNF signaling, we pretreated cells with small molecule inhibitors of necroptosis, necrostatin-1s (Nec-1s)^17^ which prevents upstream signaling of RIP1 in necroptosis, and necrosulfonamide (NSA),^50^ which prevents translocation of phosphorylated MLKL to the plasma membrane that initiates membrane permeabilization. We induced necroptosis and examined the level of mature SREBP1 using western blotting in the presence and absence of Nec-1s and NSA (Fig. S4A, ESI†). We observed a strong decrease in mature SREBP1 levels when necroptotic cells were treated with Nec-1s and NSA. These results suggest that maturation and activation of SREBP1 occur downstream of RIPK-mediated MLKL phosphorylation and subsequent translocation to the plasma membrane.

### SREBP activation exacerbates necroptosis *via* the increase in downstream VLCFAs production

**Fig. 3:**
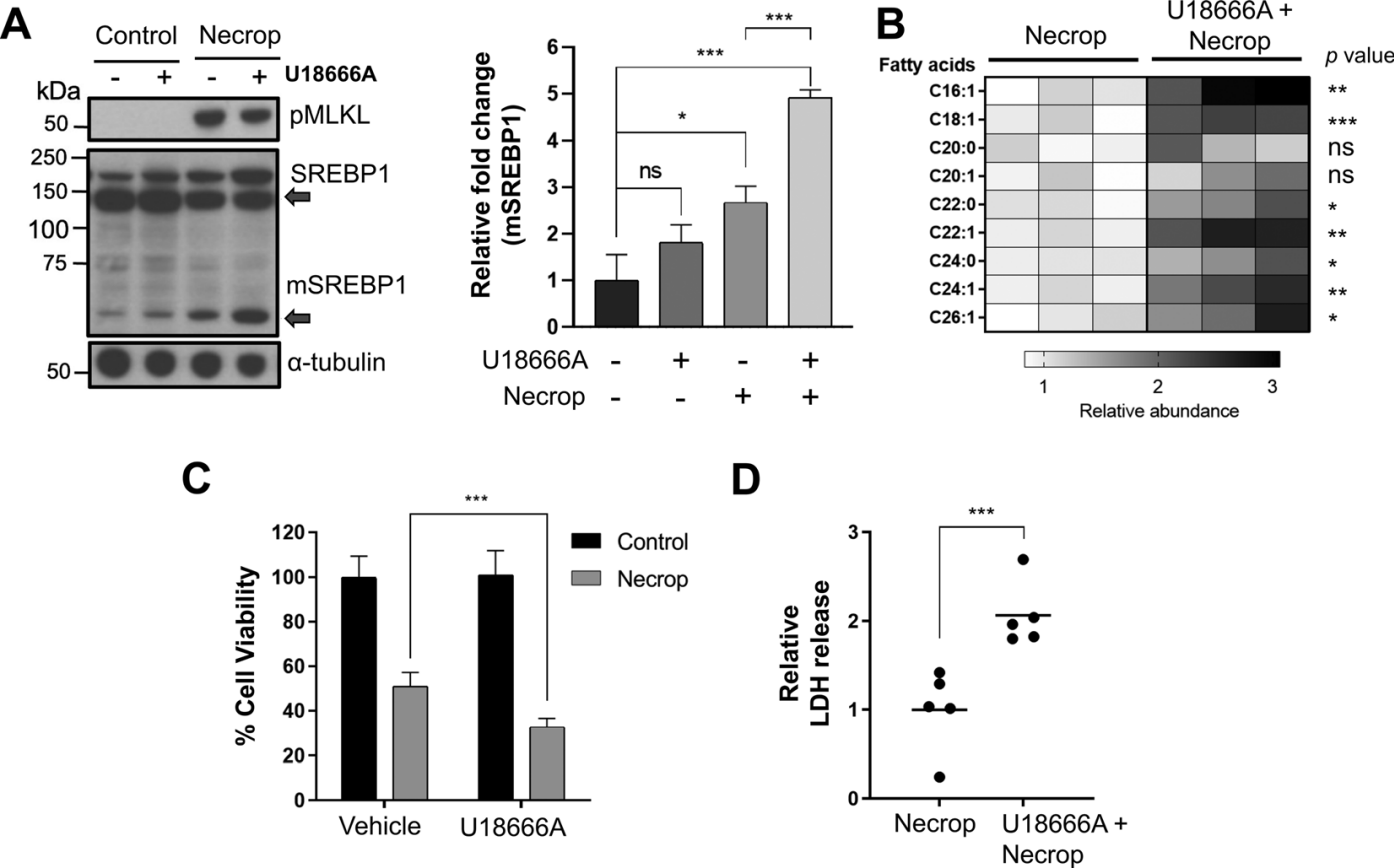
U18666A, an SREBP1 activator, contributes to VLCFA accumulation resulting in an increase in toxicity during necroptosis. (A) Western blotting showed SREBPs are further activated after U18666A pretreatment during necroptosis. HT-29 was pretreated with 3 μM of U18666A for 24 h, induced with necroptosis for 3 h. The cells were collected and prepared for western blotting experiment as whole lysate sample. The panel on the right is the quantification of mSREBP1 band intensities which shows a significant increase when pretreated with U18666A during necroptosis compared to control cells and necroptotic cells. Data represent mean ± 1 standard deviation (SD); *n* = 3. * represents *p* < 0.05, *** represents *p* < 0.001. (B) LC–MS showed further VLCFA accumulation in U18666A treated cells compared to necroptosis only cells. HT-29 was pretreated with 3 μM of U18666A for 24 h, induced with necroptosis for 3 h. The cells were collected and were subjected to lipid extraction and LC–MS analysis. *n* = 3, ns represents *p* > 0.05, * represents *p* < 0.05, ** represents *p* < 0.01, *** represents *p* < 0.001. (C) Cell viability during necroptosis decreases when SREBP1 is further activated using U18666A. HT-29 were pretreated with 3 μM of U18666A for 24 h, induced with necroptosis for 3 h, and subjected to MTT cell viability assay. Data represent mean ± 1 SD; *n* ≤ 5, *** represents *p* < 0.001. (D) Further activation of SREBP1 leads to increase in membrane permeabilization during necroptosis. HT-29 was pretreated with 3 μM of U18666A for 24 h, induced with necroptosis for 3 h, and subjected to lactate dehydrogenase release assay. Data represents mean ± 1 SD; *n* = 5, *** represents *p* < 0.001.

To study the functional involvement of SREBP1 activation in fatty acid production during necroptosis, we used a pharmacological approach. We used U18666A, an SREBP activator,^51^ to investigate whether further activation of SREBP1 *via* U18666A during necroptosis would lead to an increase in VLCFA production and hence, an increase in toxicity. We pre-treated HT-29 cells with 3 μM U18666A for 24 h, induced necroptosis, and assessed the levels of SREBP1 activation after U18666A treatment during necroptosis using Western blotting. As we expected, U18666A treatment in necroptosis activated SREBP1 as indicated by the increase in mature SREBP1 levels during necroptosis (Fig. 3(A), left panel is a representative western blot, the right panel is the quantification of mature SREBP1 levels). We then conducted targeted LC–MS-analysis to investigate if fatty acids, specifically VLCFAs were also increasing in the presence of U18666A during necroptosis. We extracted lipids from necroptotic and U18666A-treated necroptotic cells and analyzed these extracts as we described earlier.^39^ We observed a ∼3-fold increase in the levels of fatty acids, including VLCFAs that cause membrane permeabilization during necroptosis (Fig. 3(B)). The VLCFA accumulation correlates to further activation of SREBP1 observed, potentially exacerbating cell death during necroptosis. To evaluate if the accumulation of VLCFAs could be translated to an increase in necroptotic cell death and membrane permeabilization, we measured the cell viability and intracellular release of lactate dehydrogenase (LDH) of cells pretreated with U18666A in necroptosis. We indeed observed a significant decrease in cell viability (Fig. 3(C)) and a significant increase in LDH release in U18666A-treated necroptotic cells compared to necroptotic condition alone (Fig. 3(D)). Altogether, these results support that the activation of SREBP1 exacerbates necroptosis.

### Preventing SREBP activation ameliorates membrane permeabilization and rescues cells from necroptosis

**Fig. 4:**
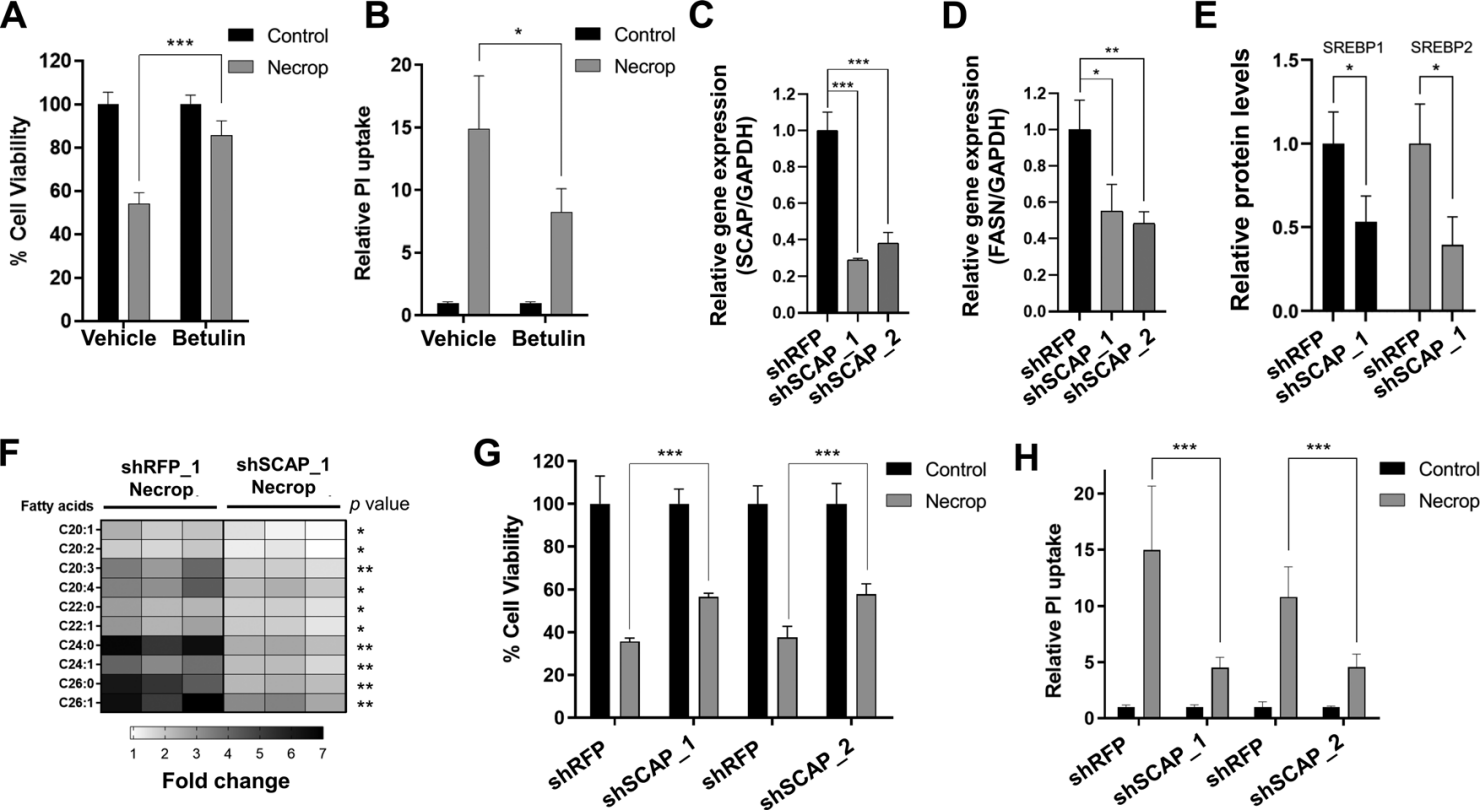
Betulin, SREBP deactivator, and shSCAP knockdown rescue cells from membrane permeabilization. (A) Cell viability during necroptosis increases when SREBP is deactivated by betulin. HT-29 was pretreated with 2 μM of betulin for 24 h, induced with necroptosis for 3 h, and subjected to MTT cell viability assay. Cell viability was normalized to the corresponding control condition. Data represent mean ± 1 standard deviation (SD); *n* = 5, *** represents *p* < 0.001. (B) Betulin prevents membrane permeabilization in necroptosis as indicated by reduced fluorescence intensity in propidium iodide uptake. HT-29 was pretreated with 2 μM of betulin for 24 h, induced with necroptosis for 3 h, and subjected to propidium iodide uptake experiment. Data represent mean ± 1 SD; *n* = 5, * represents *p* < 0.05. (C) shSCAP knockdown efficiency in HT-29 cells. Red fluorescent protein (RFP) was used as a knockdown control. shSCAP_1 and shSCAP_2 represent different shRNA constructs for shSCAP. Fold changes in the expression of *SCAP* are calculated as the ratio of the relative expression of *SCAP* compared with *GAPD*H in shSCAP and shRFP cells. Data represent mean ± 1 SD; *n* = 3. *** represents *p* < 0.001. (D) *FASN* is downregulated in shSCAP knockdown cells. Fold changes in the expression of *FASN* are calculated as the ratio of relative expression of *FASN* genes compared with *GAPDH* in shSCAP and shRFP cells. Data represent mean ± 1 SD; *n* = 3. * represents *p* < 0.05, ** represents *p* < 0.01. (E) Relative protein levels of SREBP1 and SREBP2 in shSCAP knockdown cells. shRFP and shSCAP_1 cells were collected and prepared for western blot. Quantification of SREBP1 and SREBP2 band intensities shows a significant decrease in knockdown cells compared to knockdown control. Data represent mean ± 1 SD; *n* = 3. * represents *p* < 0.05. (F) LC–MS showed lowered VLCFAs in shSCAP_1 cells during necroptosis when compared to shRFP necroptotic cells. The fold changes were calculated by normalizing them to the corresponding control condition. *n* = 3, * represents *p* < 0.05, ** represents *p* < 0.01 (G) Cell viability during necroptosis increases in shSCAP_1 and shSCAP_*_*_2 cells. shSCAP cells were induced with necroptosis for 3 h and subjected to MTT cell viability assay. Data represent mean ± 1 SD; *n* = 5, *** represents *p* < 0.001. (H) shSCAP knockdown reduces membrane permeabilization in necroptosis. Necroptosis was induced in shSCAP_1 and shSCAP_2 and PI uptake was measured. PI uptake was normalized to the corresponding control condition. Data represent mean ± 1 SD; *n* = 5, *** represents *p* < 0.001.

Our results suggest that SREBP activation might be responsible for the accumulation of VLCFAs that contribute to membrane permeability during necroptosis. We hypothesized that if this is the case, SREBP inactivation should then ameliorate cell death in necroptosis. Because compensatory effects between SREBP1 and 2 have been reported,^46–48^ we decided to target an upstream regulator of SREBP activation. We first used betulin^52–54^ which results in the degradation of both SREBPs. We pretreated cells with betulin, induced necroptosis, and measured cell viability and plasma membrane permeability during necroptosis. Betulin treatment resulted in a significant rescue from cell death during necroptosis (85% viability in betulin-treated necroptotic cells vs 50% viability in necroptotic cells, Fig. 4(A)) and restored plasma membrane permeability, measured by ∼40% decrease in propidium iodide uptake (*p* < 0.05, Fig. 4(B)). The decrease of membrane permeabilization through SREBP inactivation using betulin was consistent in U937 cell line as well (Fig. S2C, ESI†). These results support our hypothesis that SREBP inhibition reduces cell death during necroptosis.

To validate small molecule-based results, we used the lentiviral knockdown approach to deactivate SREBPs by targeting an upstream chaperone protein, SCAP, that is needed for SREBP activation.^13^ We knocked down SCAP in HT-29 cells (Fig. 4(C)) and confirmed that fatty acid biosynthesis-related target genes, fatty acid synthase (*FASN*) (Fig. 4(D)) and that precursor SREBP1 and 2 levels are depleted in the knockdown model (Fig. 4(E)). We then investigated the accumulation of fatty acids and membrane permeabilization in shSCAP cells during necroptosis. shSCAP cells showed a less profound accumulation of fatty acids in necroptosis when compared to control necroptotic cells (Fig. 4(F)) and a similar rescue in cell viability in necroptosis (Fig. 4(G)). Importantly, shSCAP cells exhibited a reduction in propidium iodide (PI) uptake during necroptosis, suggesting a decrease in membrane permeabilization as compared to control necroptotic cells (Fig. 4(H)). Overall, these results strongly support that the reducing the SREBP activity increases cell viability and restores membrane permeability during necroptosis.

### Potential mechanism of SREBP activation during necroptosis

One of the major mechanisms that control the SREBP activation is the cholesterol levels at the endoplasmic reticulum (ER).^55^ When the cholesterol level at the ER is low, SREBPs are proteolytically processed to their transcriptionally active form. However, studies have suggested other mechanisms for the activation of SREBP. One study showed that SREBP1 can be proteolytically activated by low phosphatidylcholine levels and identified phosphatidate phosphatase-1 as an activator of SREBP1.^56,57^ We analyzed phosphatidylcholine levels and found that they do not exhibit significant changes during necroptosis (Fig. S4B, ESI†). In parallel, our previous results showed no significant changes in phosphatidic acids and diacylglycerol levels during necroptosis;^39^ hence phospholipid-dependent activation of SREBP1 is not likely in necroptosis. Another study has shown peroxisome proliferator-activated receptors-α (PPARα) is involved in SREBP1 activation.^58^ Our transcriptomics results show that PPARα was downregulated in necroptosis (Table S1, ESI†*p*_adjusted_ = 0.0005), which does not support PPARα-mediated SREBP1 activation in necroptosis.

Therefore, it is possible that SREBP activation in necroptosis can be due to low cholesterol levels at the ER, either due to impaired biosynthesis or trafficking. Since we have shown that cellular cholesterol level does not change and that inhibition of cholesterol biosynthesis does not affect cell viability during necroptosis (Fig. S3C, ESI†), we investigated cholesterol localization. We used fluorescence microscopy to visualize intracellular cholesterol localization during necroptosis. We induced necroptosis, and stained cells with filipin, a fluorescent polyene molecule that binds to free cholesterols,^59^ and wheat germ agglutinin (WGA) as a membrane marker.^4,60^ We then quantified the fluorescence signal at the plasma membrane based on WGA signal. We observed that in necroptosis, cholesterol showed an increased plasma membrane localization (Fig. S4C and D, ESI†). Specifically, in necroptotic cells, filipin intensity was ∼2.5-fold higher at the plasma membrane as compared to control cells (Fig. S4D, ESI†*n* = 20, *p* < 0.001). These results suggest that cholesterol localization might be dysregulated during necroptosis and responsible for the activation of SREBP during necroptosis. Further investigations are warranted to fully establish the involvement of cholesterol in SREBP activation during necroptosis.

## Summary and conclusions

We have previously shown that fatty acid biosynthesis is activated at the transcriptional level during necroptosis and causes the accumulation of VLCFAs during this process. We also showed that VLCFA accumulation contributes to membrane damage-induced cell death in necroptosis. Here, we used transcriptomics, targeted gene expression analysis, and pharmacological perturbations, and found that SREBPs are activated during necroptosis. We showed that using an SREBP activator triggers downstream activation of lipid production and increases toxicity during necroptosis. On the contrary, inactivating SREBPs rescues necroptotic cell death and reduces membrane permeabilization. These results strongly support that SREBPs-mediated lipid production play a role in the accumulation of toxic VLCFAs during necroptosis.

Activation of *de novo* lipogenesis due to SREBP activation is related to several pathologies, including the progression of different forms of cancers.^61,62^ In these systems, increased *de novo* lipid biosynthesis provides the necessary building blocks for uncontrolled cell division.^61^ Other than potential implications in cancer therapeutics, blocking SREBP activation and decreasing lipid content *in vivo* has shown promising potential to combat metabolic diseases such as type II diabetes and atherosclerosis.^63^

Subsequent pro-inflammatory response upon membrane permeabilization is a hallmark of necroptosis. The leakage of intracellular components such as pro-inflammatory cytokines is associated with many diseases.^64,65^ One study has reported that necroptosis is activated in advanced atherosclerotic plaques and targeting the necroptosis pathway effectively reduced plaque vulnerability and lesion progression.^63^ Necroptosis also plays a complex role in cancer formation and progression. It can promote cancer metastasis and cause immunosuppression in certain types of cancers. In addition, it is well known that necroptotic cells are associated with elevated levels of inflammatory cytokines which promote cancer cell proliferation and eventual metastasis.^32^ As a result, reducing membrane permeabilization during necroptosis can play a critical role in reducing inflammatory activity in necroptosis. Along these lines, targeting SREBP activation might be a new potential mechanism to reduce membrane permeabilization due to the accumulation of fatty acids in necroptotic cells, possibly reducing the release of pro-proinflammatory molecules during this process.

## Experimental section

### Materials

HT-29 (human colorectal adenocarcinoma epithelial) cells were obtained from the American Type Culture Collection. Dulbecco's Modification of Eagle's Medium (DMEM), (Cat # 10-017-CV), Roswell Park Memorial Institute Medium (RPMI) 1640 (Cat # 15-040-CV), penicillin-streptomycin (Cat # 30-002-CI) and trypsin (Cat # 25-050-CI) were acquired from Corning. Fetal Bovine Serum (FBS) and dimethyl sulfoxide (DMSO) (Cat # 42780) were obtained from Sigma-Aldrich and Alfa Aesar, respectively. Antibodies were obtained from Abcam (rabbit monoclonal anti-phospho S358-MLKL, Cat # ab187091; rabbit polyclonal anti-SREBP1, Cat # ab28481), Cayman (rabbit polyclonal anti-SREBP2, Cat # 10007663), Millipore Sigma (mouse monoclonal anti-α-tubulin, Cat # T9026) Promega (goat anti-rabbit HRP conjugate, Cat # W4011), and Jackson Immunoresearch Lab (goat anti-mouse HRP conjugate, Cat # 115-035-174). BV6 (Cat # S7597) and simvastatin (Cat # S1792) were obtained from Selleck Chemicals, zVAD-FMK was obtained from Enzo (Cat # ALX-260-020), TNF-α was obtained from R&D Systems (Cat #210-TA/CF). Atorvastatin (Cat # 10493). BIBB 515 (Cat # 10010517), Ro48-8071 (Cat # 10006415), U18666A (Cat # 10009085), Betulin (Cat # 11041), necrosulfonamide (Cat # 20844) and filipin III (Cat # 70440) were obtained from Cayman Chemical. TAK-475 (Cat # SML2168), necrostatin-1s (Cat # 504297) and terbinafine (Cat # 78628-80-5) were obtained from Sigma Millipore. Wheat Germ Agglutinin Alexa Fluor™ 594 Conjugate (Cat # LSW1162) was obtained from Invitrogen. Protease inhibitor (Cat # A32955) were obtained from Thermo Fisher Scientific. MTT reagent (Cat # L11939) was obtained from Alfa Aesar. ProLong™ Gold Antifade Mountant (Cat # P36930) and Propidium iodide (Cat # P1304MP) were obtained from ThermoFisher. E.Z.N.A. HP total RNA kit (Cat # R6812-02) was obtained from OMEGA bio-tek. iScript Advanced cDNA Synthesis Kit (Cat # 172-5037) and ddPCR Supermix for Probes (Cat # 186-3026) were obtained from Bio-Rad. PCR primers listed in table S3 were obtained from Integrated DNA Technologies. Commercial shRNA targeting SCAP (TRCN0000296200 and TRCN0000078063) were obtained from Millipore Sigma as bacterial glycerol stock. LDH cytotoxicity assay kit (Cat # 601170) was obtained from Cayman Chemical.

### Methods

#### Cell culture and culture conditions

Human colorectal adenocarcinoma HT-29 cell (ATCC® HTB-38™, adult Caucasian female origin) were cultured at 37 °C in 5% CO_2_ atmosphere in DMEM supplemented with 10% (v/v) fetal bovine serum and 1% (v/v) penicillin/streptomycin solution. Cells were cultured for approximately 2 months and were routinely checked for mycoplasma infection. U937 lymphoblast cells were obtained as a gift from Catherine Wrona (University at Buffalo, SUNY, Division of Pulmonary, Critical Care and Sleep Medicine). U937 was maintained as suspension culture in RPMI 1640 media supplemented with 2 mM l-glutamine, 10% (v/v) fetal bovine serum and 1% (v/v) penicillin/streptomycin.

#### Inducing Necroptosis in HT-29 or U937 cells

To induce necroptosis in HT-29 cells, the cells were initially sensitized to TNF-dependent cell death pathway by SMAC mimetic BV6 (1 μM), and were co-treated with pan-caspase inhibitor zVAD-FMK (25 μM) and incubated for 30 min at 37 °C. HT-29 cells were then treated with TNF-α (10 ng mL^−1^) and were incubated for 3 h. To induce necroptosis in U937 cells, the cells were pretreated with BV6 (0.2 μM), and zVAD-FMK (25 μM) and incubated for 2 h at 37 °C. U937 cells were then treated with TNF-α (3 ng mL^−1^) and were incubated for 5 h.

#### Cell viability assays

HT-29 cells were seeded in 96-well plates with 30 000 cells per well (for 2 h pretreatment), 15 000 cells per well (for 24 h pretreatment) or 10 000 cells per well (for 48 h pretreatment. For shSCAP knockdown viability assay), 30 000 cells per well were seeded. The cells were left to attach for 20 h before pretreatment with small molecule inhibitors or induction of necroptosis. For U937 cells, 80 000 cells per well were seeded in 96-well plate and immediately undergoes pretreatment with small molecule inhibitor and/or induction of necroptosis. After the designated pretreatment time and/or necroptotic treatment, the 96-well plate was centrifuged for 2 min at 200 rcf at room temperature. The media was removed from each well and replaced with 200 μL of fresh media that contains 5 mg mL^−1^ of MTT reagent. The plate was incubated at 37 °C for 2.5 h and then centrifuged for 2 min at room temperature. 155 μL of media was removed from each well and 90 μL of DMSO was added back. The plate was then incubated at 37 °C for 10 min to solubilize the formazan crystal and was centrifuged at 1000 rcf for 2 min at room temperature. Absorbance was measured at 550 nm using Biotek Synergy HT plate reader. To calculate the percent viability of treated cells compared to control cells, the raw absorbance values of cells were subtracted from the average absorbance values of blanks. For the effect of U18666A on cell viability during necroptosis, corrected absorbance values were normalized to the average absorbance values of vehicle-treated control cells and were expressed as percentage cell viability. For the effect of betulin on cell viability during necroptosis, corrected absorbance values were normalized to the average absorbance values of each condition's treated control cells and were expressed as percentage cell viability Results are representative of at least two independent experiments, with *n* ≥ 3 each.

#### Western blotting

HT-29 cells (1 × 10^7^) were plated in 10 cm dishes for 20 h attachment. Necroptosis was induced in the cells using the method described above. For experiments with U18666A, 4 × 10^6^ cells were plated in 10 cm dishes and left to attach for 20 h. Cells were pretreated with U18666A (3 μM final concentration) or DMSO for 24 h. For the shSCAP knockdown cells, shRFP and shSCAP (approximately 3 × 10^6^ cells) were plated and left to attach for 20 h. After the cells received the pretreatment and/or induction of necroptosis, cells were scraped from the plates on ice and transferred to 15 mL centrifuge tubes. Plates were rinsed with cold 1× PBS after scraping, which was also transferred to the centrifuge tube. Cells were centrifuged for 5 min at 500 rcf at 4 °C. The supernatant was decanted and the cell pellets were washed two more times with cold PBS. The supernatant was decanted and the cell pellet was stored at −80 °C. The frozen cell pellets were thawed on ice and were lysed by re-suspending in lysis buffer (1 pellet of protease inhibitor dissolved in 10 mL of Mammalian Protein Extraction Reagent) for 45 mins on ice. The cell lysates were then centrifuged (16 000 rcf, 15 min, 4 °C) and the amount of protein was measured using Bradford Protein Assay according to the manufacturer's instructions. The samples were normalized based on the lowest protein amount and were diluted with 1 : 1 with a solution of 95% 5× loading and 5% 2-mercaptoethanol. The samples were boiled for 10 min and stored in −20 °C.

Samples were loaded and separated with sodium dodecyl sulfate polyacrylamide gel (10%) electrophoresis at 150 V. Polyvinyl difluoride (PVDF) membranes were then activated in methanol for five minutes. Once the membrane was activated, the separated proteins were transferred onto the PVDF membranes at 50 V for 2 h. After transfer, the membranes were blocked in 10% non-fat dry milk in tris-buffered saline (TBS)-Tween [10 mM tris-base, 100 mM NaCl, 0.1% Tween 20 (pH 7.5)] at room temperature for one hour. Membranes were washed three times at 10 minute intervals in TBS-Tween. The corresponding membranes were incubated with primary antibodies (1 : 500 dilutions for SREBP1; 1 : 1000 dilution for SREBP2; 1 : 1000 for pMLKL, 1 : 10 000, for α-tubulin). After the designated incubation time, the membranes were washed four times with TBS-Tween for 10 minute each time. The secondary antibodies used were 1 : 2000 anti-rabbit HRP conjugate and 1 : 1000 anti-mouse HRP conjugate. Secondary antibodies were diluted with 5% non-fat dry milk in TBS-Tween and incubated for one hour at room temperature. The membranes were then washed again with TBS-Tween three times, 10 min each time, prior to developing with Super Signal West Pico kit (Thermo Scientific).

#### Quantification of mSREBP1 and mSREBP2 bands

Quantitative analysis of Western blot images was performed by using Fiji-ImageJ software as previously described.^38^ Briefly, a frame for measurement was developed by using the rectangle tool of Fiji-ImageJ to cover the largest band of the protein of interest. The same frame size was then applied for measuring the intensity of the other protein bands. The background was also measured with the same frame to obtain background intensity measurements. For the background measurement, a region near the protein of interest was used. The measured intensities from the protein of interest and background were then inverted by deducting measurements from 255. The inverted intensities of the protein bands of interest were corrected with the inverted intensities of the background. The relative intensities were obtained by dividing the corrected intensity of mSREBP1 or mSREBP2 by the corrected intensity of the loading control (α-tubulin, *n* = 3).

#### Lentiviral knockdown of SCAP in HT-29

Knockdown of SCAP in HT-29 cells was performed similarly as described previously.^39^ Briefly, shRNA (in the pLKO.1-Puro lentiviral vector) targeting SCAP plasmid DNA (TRCN0000296200 or TRCN0000078063) was packaged into lentivirus particles in HEK293T cells *via* co-transfection with psPax2 and pCMV-VSV-G using X-tremeGENE 9 transfection reagent for 48 h. The viral particles were collected and filtered with a 0.45 μM filter. For lentiviral transfection in HT-29 cells, 5 × 10^5^ cells were plated and left to attach overnight. The following day, the media was replaced with 8 μg mL^−1^ polybrene media and incubated for 5 min after 5 min, 50 μL of shRFP (red fluorescent protein) or shSCAP viral suspension was added to each designated well and incubated for 48 h. The virus-containing media was removed and transfected cells were transferred to a new flask in 2 μg mL^−1^ puromycin-containing media. The cells were selected for 2 days and the media was changed to 1 μg mL^−1^ puromycin and were then cultured continuously in the 1 μg mL^−1^ puromycin-containing media.

#### Propidium iodide uptake

For betulin pretreatment, HT-29 cells (15 000 cells per well) were seeded in 96-well plates and were left to attach for 20 h. Betulin (final concentration 2 μM) was added for 24 h pretreatment, followed by induction of necroptosis for 3 h. For shSCAP knockdown cells, shSCAP and shRFP cells (30 000 cells per well) were seeded in 96-well plates and were left to attach for 20 h. Knockdown cells were induced with necroptosis for 3 h. After 3 h necroptosis, the plate was centrifuged for 2 min at 200 rcf at room temperature. Media in the plate was removed and added back with 200 μL propidium iodide (5 μg mL^−1^ in PBS). Cells were incubated with propidium iodide for 35 min at 37 °C. After incubation, the plate was centrifuged for 2 min at 300 rcf. The fluorescence intensity was measured using Biotek Synergy H1 microplate reader (excitation wavelength of 535 nm and emission wavelength of 625 nm). Relative fluorescence units were reported as calculated after performing blank subtraction and normalized to vehicle control or corresponding control.

#### LDH release assay

For HT-29 cells, 15 000 cells per well were seeded in a 96-well plate and left to attach for 20 h. HT-29 cells were then pretreated with 3 μM of U18666A for 24 h before inducing necroptosis. For U937 cells, 60 000 cells per well were seeded in a 96-well plate and were pretreated with 2 μM betulin immediately for 24 h before inducing necroptosis. After U18666A or betulin pretreatment, HT-29 and U937 cells were induced with necroptosis as described above. LDH release assay procedure was adapted based on the Cayman Chemical LDH cytotoxicity assay kit (Cat # 601170) with minor adjustments to the protocol. Briefly, LDH reaction was prepared as described in the manufacturer's protocol. 100 μL of the LDH reaction solution was added to a new 96-well plate. After necroptosis induction, the cell plate was centrifuged for 5 min at 400 rcf at room temperature. 100 μL of cell solution from DMSO or inhibitor-treated cells were taken out and added into the new 96 well plate containing LDH reaction solution. For blanks, 100 μL of cell solution from wells that did not receive any DMSO or small molecule inhibitor treatment, was taken and added to the LDH reaction solution. The plate was then gently shaken in New Brunswick Scientific C25KC incubator shaker (37 °C, 120 rpm, 30 min). After 30 min shaking, the plate was read at 490 nm with Biotek Synergy HT plate reader. Blank absorbance was subtracted from raw absorbance. The relative LDH release in necroptosis in HT-29 cells was calculated by normalizing absorbance to necroptotic control. The relative LDH release in necroptosis in U937 cells was calculated by normalizing to the corresponding control and then normalized to necroptotic control.

#### Gene expression measurements

HT-29 cells (approximately 1.5× 10^6^ per well) were seeded onto a 6-well plate and were left to attach for 20 h. Cells were induced with necroptosis for 3 h and 5 h or DMSO as control (*n* = 3). For the shSCAP knockdown cells, shRFP and shSCAP (approximately 1.5 × 10^6^ cells per well) were plated in 6-well plates (*n* = 3) and left to attach for 20 h. After induction of necroptosis or attachment, cells were collected and the total RNA was extracted according to E.Z.N.A HP Total RNA kit manufacturer's protocol. BioRad's Droplet Digital PCR: QX200 System was used to convert to cDNA with iSCript cDNA synthesis kit, and to amplify and quantify cDNA. Reaction mixtures including primers, probes, and cDNA were prepared with ddPCR Supermix for Probes (no dUTP) according to the manufacturer's instructions. An automated droplet generator was used to create water–oil emulsion droplets from the reaction mixtures. A QX200 droplet reader was used to quantify HEX and FAM fluorescence in 20 000 droplets per sample. BioRad Quantasoft software was used for data analysis. The relative expression was calculated based on the ratio between the gene of interest and the housekeeping gene (*HPRT1 or GAPDH*). The foldchange was calculated by normalizing the relative expression in necroptosis to the DMSO control. For shSCAP knockdown model, the foldchange was calculated by normalizing the relative expression in shSCAP knockdown cells to shRFP cells. The primer sequences are given in Table S3 (ESI†).

#### Lipid extraction and LC–MS data acquisition for fatty acids and PC analysis

Procedures for lipid extraction and LC–MS methods were adapted from a previous study (Parisi *et al.*, 2017). Cell pellets were thawed on ice and resuspended in 1 mL cold PBS. A 30 μL aliquot of this cell suspension was taken and added to an equal volume of lysis buffer on ice for 1 h. The protein concentration of the sample was then determined using Bradford Protein Assay. The remaining 970 μL cell suspension was transferred to a Dounce tissue homogenizer to which 1 mL of cold methanol and 2 mL cold chloroform were added. The mixture was homogenized with 30 strokes and then transferred to a 2-dram glass vial. The vials were centrifuged (500 rcf, 10 min, 4 °C) and the chloroform layer was transferred to a 1-dram glass vial. From the chloroform layer, 1.2 mL was further transferred into a new 1-dram to ensure an equal volume was taken from each sample. The chloroform was removed by rotary evaporation and the dried lipids were stored at −80 °C until ready to be analyzed. Lipids were resuspended in chloroform with volume normalized to protein concentration.

LC–MS analyses were performed using an Agilent Infinity 1260 HPLC/Agilent 6530 Jet Stream ESI-QToF-MS system. Reverse phase gradient elution chromatography was used for separations. Mobile phase A was composed of 95% water and 5% methanol. Mobile phase B was composed of 60% isopropanol, 35% methanol, and 5% water. For positive ionization mode analyses, 0.1% (v/v) formic acid and 5 mM ammonium formate were added to both mobile phases to improve ionization. For negative ionization mode analyses, 0.1% (w/v) ammonium hydroxide was added instead. LC gradient started with 5 min of 0% B at 0.1 mL min^−1^, then the flow rate was increased to 0.5 mL min^−1^. The mobile phase gradient was ramped from 0% B to 100% B over 60 min, maintained at 100% B for 7 min, then switched to 0% B for 8 min. For positive ionization mode analyses, a Luna C5 column was used as the stationary phase along with a C5 guard cartridge. For negative ionization mode analyses, a Gemini C18 column was used along with a C18 guard cartridge. A DualJSI fitted electrospray ionization (ESI) source was used for MS analysis with a capillary voltage of 3500 V and fragmentor voltage of 175 V. Drying gas temperature was 350 °C with a flow rate of 12 L min^−1^. Data were collected using an *m*/*z* range of 50–1700 in extended dynamic mode. Tandem mass spectrometry data were collected using the following collision energies: 15, 35, 55, and 75 eV for each *m*/*z*. Abundances were obtained from peak integration of the extracted ion chromatograms. Fatty acids were analyzed in negative mode as[M−H]^−^ adducts, and PC were analyzed in positive mode as [M+H]^+^ adducts. For fatty acids or phosphatidylcholine that were detected in blank injections, blank subtractions were carried out.

#### Analysis of fatty acids in shSCAP during necroptosis

shRFP and shSCAP (8 × 10^6^ cells) were plated in 10 cm dishes for ∼20 h attachment. The cells were added BV6 (1 μM), and zVAD-FMK (25 μM) and incubated for 30 min at 37 °C. Necroptosis was induced using TNF-α (10 ng mL^−1^ final) for 3 h. The cells were then collected on ice, centrifuged, washed with PBS, and then stored at −80 °C until lipid extraction. Lipid extraction and fatty acid analysis in LC–MS were performed as described above. Abundances of different fatty acids were obtained from peak integration and blank subtraction was carried out. Fold changes of fatty acids in shRFP necroptosis condition were calculated by dividing the abundance of the fatty acid in shRFP necroptosis by the average abundance of shRFP control. Fold changes of fatty acids in shSCAP necroptosis conditions were calculated by dividing the abundance of the fatty acid in shSCAP necroptosis by the average abundance of shSCAP control.

#### Analysis of fatty acids and phosphatidylcholines (PCs) in U18666A treated cells during necroptosis

HT-29 cells (4 × 10^6^ cells) were plated on 10 cm dishes for ∼20 h attachment. Cells were pretreated with U18666A (3 μM final concentration) or DMSO for 24 h. Necroptosis was induced using TNF-α (2 ng mL^−1^ final, with BSA as carrier protein) for 3 h. After 3 h, the cells were collected on ice, centrifuged, washed with PBS, and then stored at −80 °C until lipid extraction. Lipid extraction and lipid analysis in LC–MS was performed as described above. Abundances of different fatty acids and PC were obtained from peak integration and blank subtraction was carried out. Relative abundance of the fatty acids in necroptotic cells or U18666A treated necroptotic cells were calculated by dividing the abundances of the lipids by the average abundance of necroptotic cells. Relative abundance of PCs were calculated by dividing the abundances of the lipids by the average abundance of control cells.

#### Analysis of cholesterol during necroptosis

HT-29 cells (1 × 10^7^) were plated in 10 cm dishes for ∼20 h attachment. The cells were added BV6 (1 μM), and zVAD-FMK (25 μM) and incubated for 30 min at 37 °C. Necroptosis was induced using TNF-α (10 ng mL^−1^ final) for 3 h. The cells were collected and lipids were extracted as described above. Briefly, cell pellets were thawed on ice and resuspended in 1 mL cold PBS. A 30 μL aliquot of this cell suspension was taken for protein measurement using a Bradford Protein Assay. The remaining suspension was transferred to a Dounce tissue homogenizer to which 1 mL of cold methanol and 2 mL cold chloroform were added. The mixture was homogenized with 30 strokes and then transferred to a 2-dram glass vial. The vials were centrifuged (500 rcf, 10 min, 4 °C) and the chloroform layer was transferred to a 1-dram glass vial. The methanol: PBS layer was extracted again by adding another 2 mL of chloroform. The mixture was vortexed for 5 s, 3 times, and centrifuged (500 rcf, 10 min, 4 °C). The chloroform layer is transferred into the 1-dram vial, from which 3 mL of chloroform layer was further transferred into a new 1-dram vial. The chloroform layer was dried using Reacti-Vap™ Evaporators. The samples were reconstituted in chloroform with volumes normalized according to protein concentration.

LC–MS analyses were performed similarly as described above. Briefly, the analysis of cholesterol was carried out using an Agilent Infinity 1260 HPLC/Agilent 6530 Jet Stream ESI-QToF-MS system. 5 μL of the sample was injected for each analysis. Reverse phase gradient elution chromatography was used for separations. Cholesterol detection was carried out in positive ionization mode. Mobile phase A was composed of 95% water and 5% methanol. Mobile phase B was composed of 60% isopropanol, 35% methanol, and 5% water. For positive ionization mode analyses, 0.1% (v/v) formic acid and 5 mM ammonium formate were added to both mobile phases to improve ionization. LC gradient started with 5 min of 0% B at 0.1 mL min^−1^. The gradient was ramped to 100% B over 60 min, and maintained at 100% B for 7 min. For positive ionization mode analyses, a Luna C5 column was used as the stationary phase along with a C5 guard cartridge. A DualJSI fitted electrospray ionization (ESI) source was used for MS analysis with a capillary voltage of 3500 V and fragmentor voltage of 175 V. Drying gas temperature was 350 °C with a flow rate of 12 L min^−1^. The abundance of cholesterol was obtained from peak integration and blank subtraction was carried out. Fold changes were calculated by dividing the abundance of cholesterol in necroptotic cells by the average abundance of control cells.

#### Filipin staining for cholesterol localization during necroptosis

HT-29 cells (15 × 10^4^) were seeded onto glass coverslips in 24-well plates and were left to attach for 20 h. Necroptosis was induced using 2 ng mL^−1^ TNF-α for 3 h. After induction of necroptosis, the coverslips were washed three times with PBS and the cells were fixed using 3.7% paraformaldehyde for 30 min at room temperature. Coverslips were washed with PBS and the cells were stained with Filipin (final concentration 10 μg mL^−1^ filipin, which was diluted 1 : 50 in PBS from 0.5 mg mL^−1^ filipin stock) overnight at 4 °C. The following day the coverslips were washed three times with PBS, and were stained with WGA-594 (1 : 2000 dilution in 2% BSA-TBS) for 1 h at room temperature. The coverslips were subjected to three PBS washes and were mounted on glass slide using Prolong Gold Antifade Reagent. Images were acquired on a LSM710 confocal microscope (Carl Zeiss, Oberkochen, Germany). For quantification of filipin intensity localization in control and necroptotic cells, Fiji-imageJ software was used. Quantification of filipin intensity for control and necroptotic cells is done in the following steps: (1) the sum of projection was obtained. A straight line (line width/thickness = 1, line size is 32.38 μm) across the cell was drawn using the line scan feature in Fiji (a total of 10 cells were chosen for quantification) (2) Filipin and WGA-594 intensity profiles were obtained according to the line drawn across the cell. The filipin signal at the plasma membrane was obtained based on the intensity of the plasma membrane marker, Alexa 564 WGA. Cytoplasmic filipin signal was obtained the same way. (3) The ratio of the filipin signal at the plasma membrane and cytoplasm was reported was used to assess cholesterol localization.

#### Transcriptomics

Samples were processed by TrueSeq using standard library preparation workflow and single-read sequenced *via* Illumina HiSeq 2500. We conducted FASQC and MultiQC to test the quality of reads Quality control of paired reads was performed using FastQC and MultiQC.^66^ We used Kallisto to align the reads to Hg19 and quantify expression of each gene (ref. 67). We then used DESeq2 to compare the transcript abundances for each gene between control and target samples and identify differentially expressed genes using the Benjamini and Hochberg false discovery rate (FDR) corrected adjusted *P* value, *P* < 0.05.^68^ We provide the gene expression levels and the results of comparative analysis in Table S1 (ESI†). Raw RNAseq dataset can be accesed at Short Read Archive (SRA, https://www.ncbi.nlm.nih.gov/sra, accession number: PRJNA935316).

#### Statistics

All statistical analysis for the western blot quantifications, cell viability experiments, LC–MS lipid abundance, and ddPCR were performed using unpaired Student's *t* test. *p* values and numbers of replicates in all figures are indicated in figure legends, where ***p < 0. 001, **p < 0.01, *p < 0.05, and ns is *p* > 0.05. SD represents standard deviation. For transcriptomic analysis, the Log_2_ fold changes, *p* and *p*_adjusted_ values (calculated by Wald test for multiple hypotheses) were calculated using DESeq2.

## Author contributions

Experiments were designed by D. L., L. R. P., O. G., and G. E. A.-G. The experiments were conducted by D. L. and L. R. P. Transcriptomics analysis was done by O. G. The manuscript was written by D. L., L. R. P., O. G., and G. E. A.-G. The study was directed by G. E. A.-G.

## Conflicts of interest

The authors declare no conflict of interest.

## Supplementary Material

CB-004-D2CB00172A-s001

CB-004-D2CB00172A-s002

CB-004-D2CB00172A-s003

CB-004-D2CB00172A-s004
