# Supplementary material for: SREBP activation contributes to fatty acid accumulations in necroptosis
Source: RSC Chem Biol. 2023 Feb 13;4(4):310–22. doi: 10.1039/d2cb00172a (PMC10074566; doi:10.1039/d2cb00172a)
Supplement: CB-004-D2CB00172A-s001 [file CB-004-D2CB00172A-s001.pdf]

Electronic Supplementary Material (ESI) for RSC Chemical Biology.
This journal is © The Royal Society of Chemistry 2023

Supporting Information for

SREBP activation contributes to fatty acid accumulations in necroptosis

Daniel Lu[1], Laura R. Parisi[1], Omer Gokcumen[2], G. Ekin Atilla-Gokcumen[1,*]

[1]Department of Chemistry, University at Buffalo, The State University of New York (SUNY), Buffalo, NY 14260, USA

[2]Department of Biological Sciences, University at Buffalo, The State University of New York (SUNY), Buffalo, NY 14260, USA

*corresponding author (ekinatil@buffalo.edu)

Table S1 (Excel file). Results of transcriptomic data. Transcripts detected in samples from control and necroptotic cells are reported.
This table is provided as a separate spread sheet. The spreadsheet contains two tabs:
The first tab ("raw read depths") contains all genes detected in control and necroptotic cells:
Column A shows the ensemble gene ID for detected transcripts.
Columns B-D show the values of read depth of transcripts in control cells (n= 3)
Columns E-G show the values of read depth of transcripts in necroptotic cells (n= 3)
Column H shows the HUGO gene nomenclature (hgnc_symbol) for identified transcript
Column I shows the description of hgnc_symbol
Column J shows the Log_2 fold change during necroptosis (with respect to control) for each identified transcript
Column K shows the *p* value for each identified transcript during necroptosis (with respect to control)
Column L shows the $p_{adjusted}$ value for each identified transcript during necroptosis (with respect to control)

The second tab ("all necrop padj<0.05") contains all genes detected in control and necroptotic cells that have $p_{adjusted}$ value < 0.05 during necroptosis:
Column A shows the ensemble gene ID for detected transcripts.
Columns B-D show the values of read depth of transcripts in control cells (n=3)
Columns E-G show the values of read depth of transcripts in necroptotic cells (n =3)
Column H shows the HUGO gene nomenclature (hgnc_symbol) for identified transcript
Column I shows the description of hgnc_symbol
Column J shows the Log_2 fold change during necroptosis (with respect to control) for each identified transcript
Column K shows the *p* value for each identified transcript during necroptosis (with respect to control)
Column L shows the $p_{adjusted}$ value for each identified transcript during necroptosis (with respect to control)

Table S2 (Excel file). Results of transcriptomic data. Lipid related transcripts changing significantly in necroptotic cells compared to control are reported.
This table is provided as a separate spread sheet.

Column A shows the ensemble gene ID for detected transcripts.
Columns B-D show the values of read depth of transcripts in control cells (n= 3)
Columns E-G show the values of read depth of transcripts in necroptotic cells (n= 3)
Column H shows the HUGO gene nomenclature (hgnc_symbol) for identified transcript
Column I shows the description of hgnc_symbol
Column J shows the Log_2 fold change during necroptosis (with respect to control) for each identified transcript
Column K shows the *p* value for each identified transcript during necroptosis (with respect to control)
Column L shows the $p_{adjusted}$ value for each identified transcript during necroptosis (with respect to control)

Gene	Primer	Sequence	Identifier
ELOVL7	sense	5’-GCA ATC CTC CAT GAA AAA GAA CT-3’	Hs.PT.58.40 27298
	antisense	5’-CCA GCC TAC CAG AAG TAT TTG TG-3’	
FASN	sense	5’-GCA GTT CAC GGA CAT GGA-3’	Hs.PT.58.39 640679
	antisense	5’-CTG GTG GCT CTT GAT GAT CAG-3’	
MLYCD	sense	5'-GAT GGA ATA AAA GAT CGC AGC A-3'	Hs.PT.58.39 208007
	antisense	5'-TTT GCA CGT GGC ACT GA-3'	
HMGCR	sense	5'-CTG ACA TGC AGC CAA AGC-3'	Hs.PT.58.41 105492
	antisense	5'-GTT TAC CCT CGA TGC TCT TGT-3'	
HMGCS1	sense	5'-GCC TTC TCC ACA TCT CTA TCA AAG-3'	Hs.PT.58.27 368554
	antisense	5'-CTC GGA TGT TGC TGA ATG ACT-3'	
MCEE	sense	5'-ATG TGC TCC TAT TTT GAC CTC T-3'	Hs.PT.58.29 57789
	antisense	5'-GCA GAA AAA CAA GGC TGG AG-3'	
PPARD	sense	5'-GCC ACT GTG TGA GTA TCA CG-3'	Hs.PT.58.38 472006
	antisense	5'-GGG AAA AGT TTT GGC AGG AG-3'	
LIAS	sense	5'-TTG TCC TAA AGT CAA GCA GTC T-3'	Hs.PT.58.19 178010
	antisense	5'-CGT GTA CTG AAA CAT GCC AAG-3'	
OXSM	sense	5'-GCA GCA GCC ATA CAC CTT A-3'	Hs.PT.58.38 451856
	antisense	5'-AGT ATC CAC AGC CTG TAC CA-3'	
INSIG1	sense	5'-AAC GAT CAA ATG TCC ACC AAA G-3'	Hs.PT.58.25 883577
	antisense	5'-CAT TAA CCA CGC CAG TGC T-3'	
ACACA	*sense*<br>*antisense*	5’-GTA CAT CGC TGA CAC TAG CTA C-3’<br>5’-CTG CCC ACA TCT CAT CCA AA-3’	Hs.PT.56a.5 13712.g
SCAP	*sense*<br>*antisense*	5’-CAC GAC AGA AAG AGA CAG AA-3’<br>5’-GAG AGC TGG TCC ATC ATG AAG-3’	Hs.PT.58.45 442299
HPRT1	sense	5’-TTG TTG TAG GAT ATG CCC TTG A-3’	Hs.PT.58v.4 5621572
	antisense	5’-GCG ATG TCA ATA GGA CTC CAG-3’	
GAPDH	*sense*<br>*antisense*	5’-ACA TCG CTC AGA CAC CAT G-3’<br>5’-TGT AGT TGA GGT CAA TGA AGG G-3’	Hs.PT.39a.2 2214836

Table S3. Sequences of primers used for droplet digital PCR.

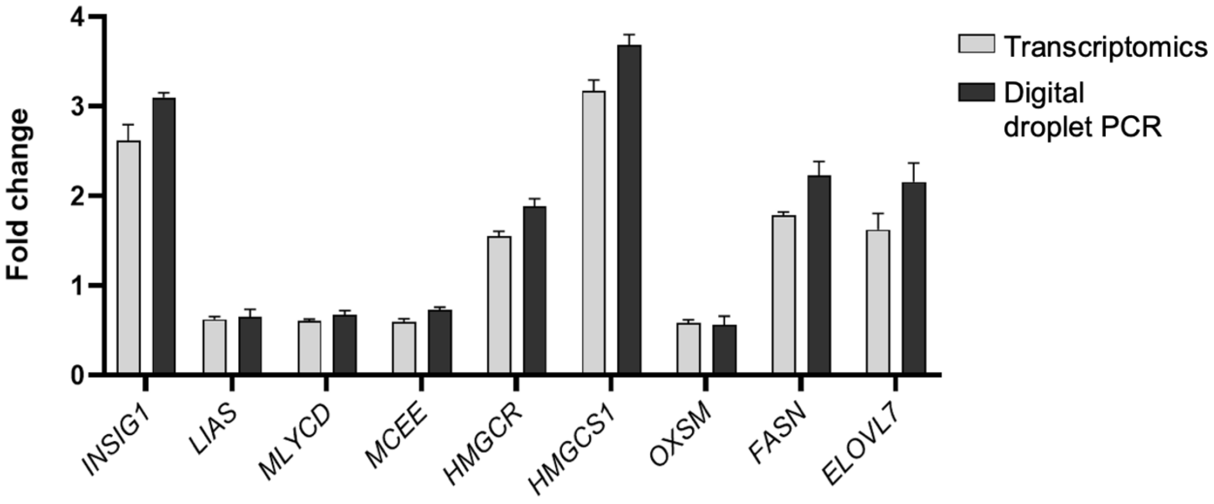

Figure S1. Validation of transcriptomics results and SRBEP activation in other cell lines. **(A)** Validation of transcriptomic results via comparison of fold changes obtained from transcriptomics dataset and from independently measured samples by digital PCR experiments. Data represents mean ± 1 SD; n= 3. Gene abbreviations: insulin induced gene 1, *INSIG1*; Lipoic Acid Synthetase, *LIAS*; Malonyl-CoA Decarboxylase, *MLYCD*; Methylmalonyl-CoA Epimerase, *MCEE*; 3-hydroxy-3-methylglutaryl-CoA reductase, *HMGCR*; 3-hydroxy-3-methylglutaryl-CoA synthase 1, *HMGCS1*; squalene epoxidase, *SQLE*; 3-Oxoacyl-ACP Synthase, Mitochondrial, *OXSM*; fatty acid synthase, *FASN*; *ELOVL7*, Elongation of Very Long Chain Fatty Acids Protein 7.

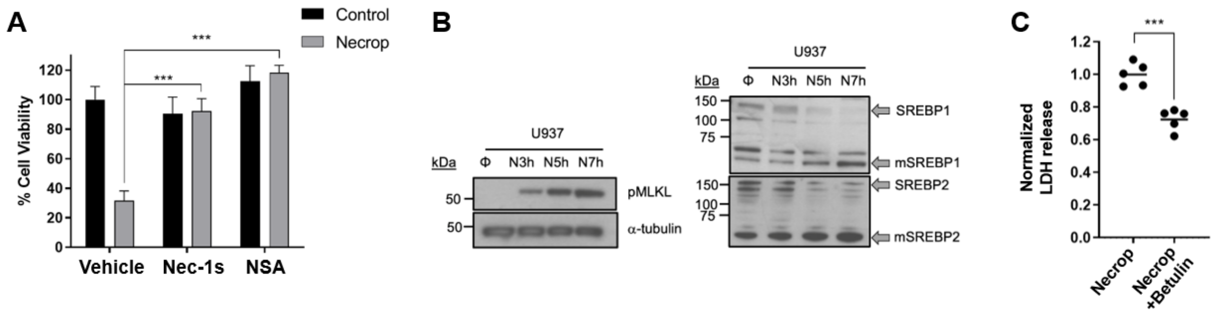

Figure S2. (A) U937 cells undergo TNF-induced necroptosis. U937 cells were plated and pretreated with necroptosis inhibitor (1 μM Nec-1s or NSA) for 2 h. Cells were sensitized towards necroptosis via pretreatment of 0.2 μM of BV6 and 25 μM zVAD-fmk for 2 h. Necroptosis was induced via the addition of 3 ng/mL TNF-α for 5 h. Cells were then subjected to MTT cell viability assay. Data represent mean ± 1 SD; n = 5, *** represents $p < 0.001$. **(B)** Western blot analysis showed SREBPs to be also activated in necroptosis similar to HT-29. U937 cells were induced with necroptosis for 3 h, 5 h, and 7 h. The whole lysate samples were prepared and analyzed. Φ represents DMSO control, N3h represents 3 h necroptosis, N5h represents 5 h necroptosis and N7h represents 7 h necroptosis. (**C**) Deactivation of SREBP via botulin treatment reduces LDH release in necroptotic U937 cells. U937 cells were seeded and pretreated with 2 μM betulin for 24 h. Cells were induced necroptosis for 5 h and subjected to LDH release assay. The relative LDH release in necroptosis in U937 cells was calculated by normalizing absorbance to the corresponding control and then normalized to the necroptotic condition. Data represent mean ± 1 SD; n = 5, *** represents $p < 0.001$.

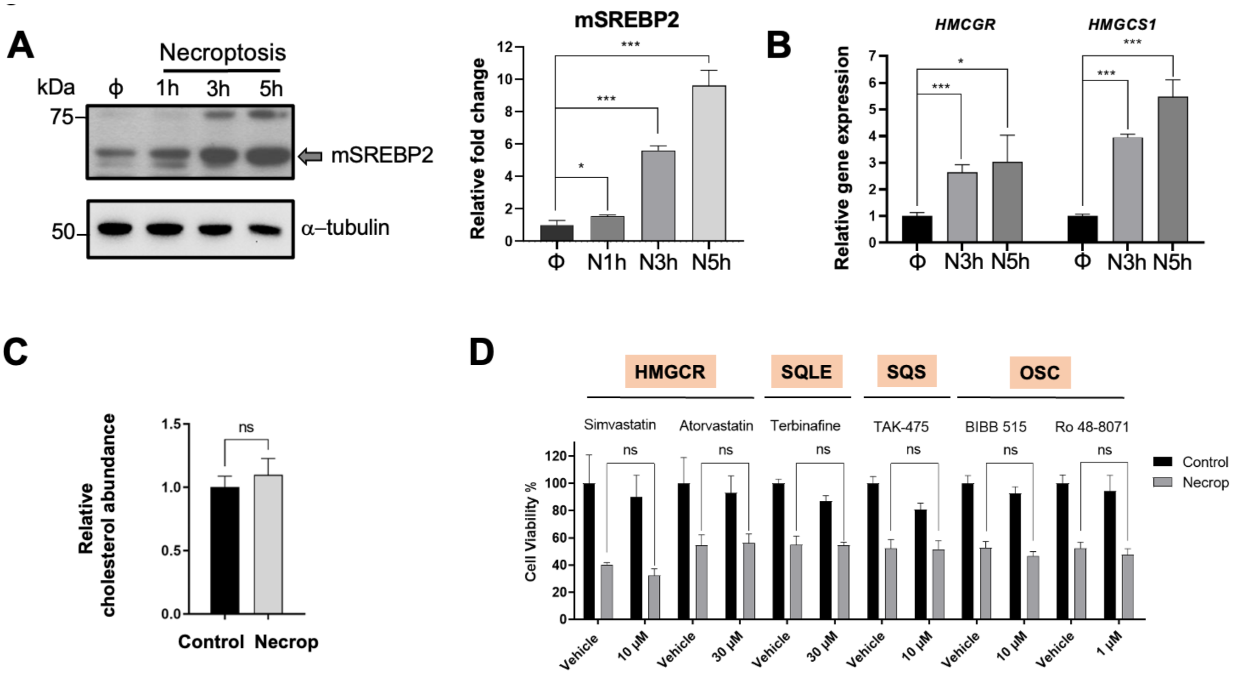

Figure S3. (**A**) Western blot analysis for the time-dependent increase in SREBP2 activation during necroptosis. Cells were induced with necroptosis for 1 h, 3 h, and 5 h. The whole lysate samples were prepared and analyzed. Mature SREBP2 (mSREBP2) also increases time-dependently compared to control during necroptosis just like SREBP1. The right panel shows the quantification of mSREBP2 band intensities. There is a significant increase in activation during necroptosis compared to control cells. Data represent mean ± 1 SD; n= 3. * represents $p < 0.05$, *** represents $p < 0.001$. Φ represents DMSO control, N1h represents 1 h necroptosis, N3h represents 3 h necroptosis, N5h represents 5 h necroptosis (**B**) SREBP2 target genes are upregulated during necroptosis. Fold changes in expression of *HMGCR* and *HMGCS1*, are calculated as the ratio of relative expression of each gene compared with *HPRT1* in necroptotic and control cells. Φ represents DMSO control, N3h represents 3 h necroptosis, N5h represents 5 h necroptosis. Data represent mean ± 1 SD; n= 3. * represents $p < 0.05$, *** represents $p < 0.001$. (**C**) LC-MS showed cholesterol level during necroptosis does not change. Data represent mean ± 1 SD; n= 3. ns represents $p > 0.05$. (**D**) Targeting the cholesterol biosynthesis pathway using small molecule inhibitors did not affect the necroptosis phenotype. HT-29 cells were plated and pretreated either with simvastatin, terbinafine, TAK-475, BIBB 515, or Ro 48-8071 for 24 h, atorvastatin for 48 h. After pretreatment cells were induced with necroptosis for 3h and subjected to an MTT cell viability assay. Data represent mean ± 1 SD; n ≤ 5, ns represents $p > 0.05$. Enzyme abbreviations: 3-hydroxy-3-methylglutaryl-CoA reductase, HMGCCR; squalene epoxidase, SQLE; 3-Oxoacyl-ACP synthase; squalene synthase, SQS; 2,3-oxidosqualene cyclase, OSC.

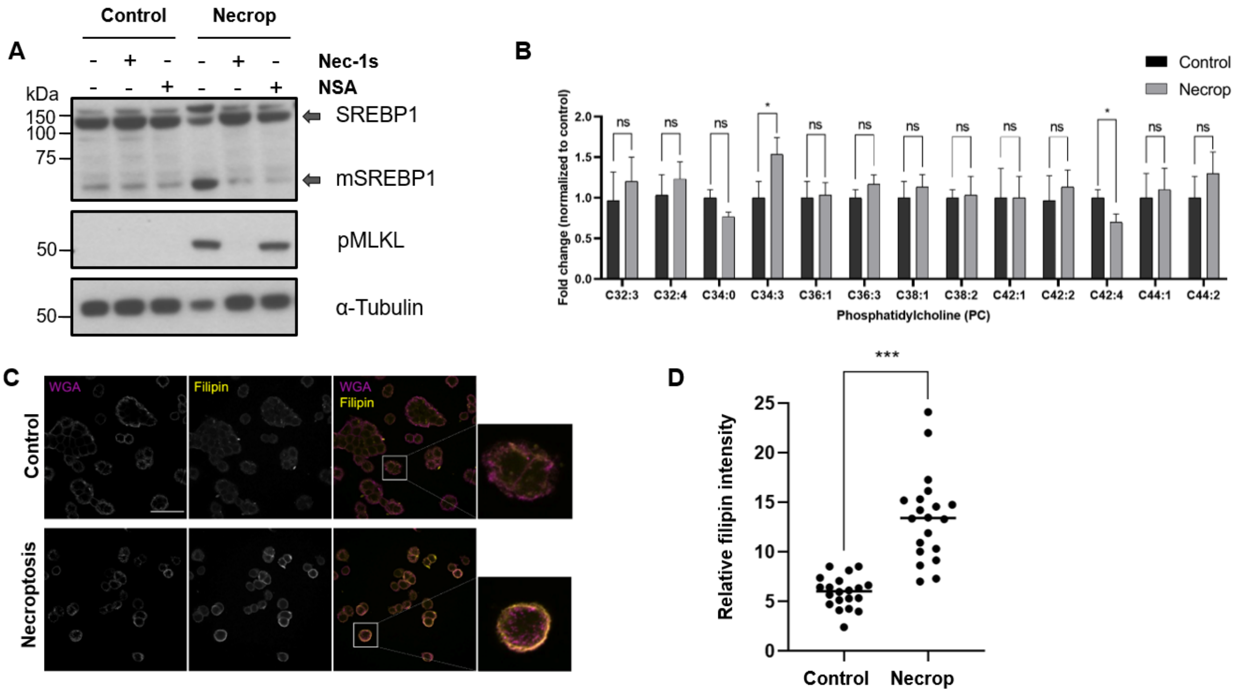

Figure S4. (**A**) HT-29 were pretreated with either 1 μM Nec-1s or 1 μM NSA and induced necroptosis. Cells were collected and prepared for western blot analysis. Activation of SREBP1 decreased for Nec-1s or NSA-treated cells during necroptosis when compared to necroptosis alone. **(B)** Phosphatidylcholine (PC) levels during necroptosis. The relative abundance of PC species (normalized to control) showed no significant changes in necroptotic cells compared to control cells alone. Data represents mean ± 1 SD; n= 3, ns represents not significant, * represents $p < 0.05$. (**C**) Fluorescence microscopy images showed cholesterol localization at the plasma membrane during necroptosis. Cells were stained with filipin which binds to free cholesterol. Representative images from at least three experiments are shown. To illustrate changes in the plasma membrane more clearly, magnified images are shown next to the merged images. The white scale bar represents 50 μm. (**D**) Quantitative analysis of cholesterol localization during necroptosis. One representative image from the control or necroptotic condition was selected for quantification (n = 20, *** represents $p < 0.001$, see supporting information method details for the quantification of filipin).
